# Identification and profiling of miRNAs during herbivory reveals jasmonate-dependent and -independent patterns of accumulation in *Nicotiana attenuata*

**DOI:** 10.1186/1471-2229-12-209

**Published:** 2012-11-07

**Authors:** Tohir A Bozorov, Ian T Baldwin, Sang-Gyu Kim

**Affiliations:** 1 Department of Molecular Ecology, Max Planck Institute for Chemical Ecology, Hans-Knöll-Straβe 8, Jena, D-07745, Germany

**Keywords:** Anti-herbivore defense, Jasmonate, *Manduca sexta*, miRNA, *Nicotiana attenuata*, tasiRNA

## Abstract

**Background:**

Plant microRNAs (miRNAs) play key roles in the transcriptional responses to environmental stresses. However, the role of miRNAs in responses to insect herbivory has not been thoroughly explored. To identify herbivory-responsive miRNAs, we identified conserved miRNAs in the ecological model plant *Nicotiana attenuata* whose interactions with herbivores have been well-characterized in both laboratory and field studies.

**Results:**

We identified 59 miRNAs from 36 families, and two endogenous *trans*-acting small interfering RNAs (tasiRNA) targeted by miRNAs. We characterized the response of the precursor and mature miRNAs to simulated attack from the specialist herbivore *Manduca sexta* by quantitative PCR analysis and used ir-*aoc* RNAi transformants, deficient in jasmonate biosynthesis, to identify jasmonate-dependent and -independent miRNA regulation. Expression analysis revealed that groups of miRNAs and tasiRNAs were specifically regulated by either mechanical wounding or wounding plus oral secretions from *M. sexta* larvae, and these small RNAs were accumulated in jasmonate-dependent or -independent manners. Moreover, cDNA microarray analysis indicated that the expression patterns of the corresponding target genes were correlated with the accumulation of miRNAs and tasiRNAs.

**Conclusions:**

We show that a group of miRNAs and tasiRNAs orchestrates the expression of target genes involved in *N. attenuata*’s responses to herbivore attack.

## Background

A group of non-coding small RNAs (smRNAs) plays an important role in transcript regulation by binding to their target sequences, resulting in transcriptional degradation, transcriptional or translational inhibition of the targets [1-5]. The smRNAs are classified into two major classes: microRNAs (miRNAs), and small interfering RNAs (siRNAs). Primary transcripts of miRNAs are processed into precursors of miRNAs that form secondary stem-and-loop structures, which are processed by the ribonuclease DICER-like 1 (DCL1) into miRNA/miRNA* duplexes, which are subsequently incorporated into the RNA induced silencing complex (RISC) [5-7]. The siRNAs are further classified into *trans* acting siRNAs (tasiRNAs), chromatin-associated *cis* acting siRNAs, and natural antisense siRNAs, based on their biogenesis [8-10]. Biogenesis of tasiRNAs is regulated by miRNAs, which direct cleavage of primary tasiRNA (*TAS*) transcripts encoding tasiRNAs, resulting in second-strand RNA synthesis by RNA-dependent RNA polymerases (RdRs). The double-stranded RNAs are diced by DCL4 to generate tasiRNAs in *Arabidopsis thaliana*[9-11].

Plant miRNAs and siRNAs are involved in several developmental processes [12]: embryogenesis [13], organ polarity [14], leaf formation [15], root development [11,16], phytohormone signaling [17,18], and flowering time [19]. Plant defense signaling is also regulated by miRNAs in response to different abiotic stresses [20,21] including heat, cold, drought [22,23], and UV-B radiation [24]. For example, *A. thaliana* miR399 (Ath-miR399), induced during phosphate starvation, targets the ubiquitin-conjugating E2 enzyme involved in phosphate uptake from the soil [25]. Under drought stress, Ath-miR159 regulates *MYB33* and *MYB101* transcription factors, which activate abscisic acid responses during seed germination [26]. Ath-miR398 regulates Cu/Zn-superoxide dismutase genes, which detoxify superoxide radicals [27]. A recent study reported that several miRNAs are induced upon mechanical wounding in tobacco leaves and roots [28].

Plant miRNAs are also involved in biotic interactions. Ath-miR393 is induced by flagellin-derived PAMP peptide 22, and targets the F-box protein and transport inhibitor response 1, which plays a key role in antibacterial responses [29]. Ath-miR160, Ath-miR167, and Ath-miR825 are induced in response to infection by *Pst* DC3000 hrcC [20], and *A. thaliana* and *Nicotiana tabacum* plants infected by TYMVp69 virus accumulate high levels of miR156, miR160, and miR164 [30,31]. Plant miRNAs are also involved in beneficial interactions with bacteria: miR482, miR1512, and miR1515 play a role during rhizobial infection in *Glycine max* nodulation with *Bradyrhizobium japonicum*[32]. However, little is known about the role of plant miRNAs in the response to insect herbivores.

The wild tobacco *Nicotiana attenuata* and its herbivore community have become an ecological model system for the study of plant-herbivore interactions. During attack by insect herbivores, *N. attenuata* rapidly induces jasmonate-mediated defense responses, which reconfigure primary and secondary metabolism [33,34]. Jasmonates comprise jasmonic acid (JA), its derivatives and conjugates; the jasmonates and in particular, the active hormone jasmonoyl-isoleucine (JA-Ile) regulate most defenses against chewing herbivores [35]. Fatty acid amino acid conjugates (FACs) in oral secretion (OS) from larvae of the specialist herbivore, *Manduca sexta*, trigger jasmonate-mediated direct and indirect defenses in *N. attenuata*, such as nicotine accumulation, proteinase inhibitor production, diterpene glycoside biosynthesis, and emission of green leaf volatiles [36-38]. Transgenic plants impaired in jasmonate biosynthesis or signaling show increased susceptibility to herbivory in both glasshouse and field studies [35,39-41].

OS-elicitation dramatically changes the smRNA population in *N. attenuata*[42], and two major components of the smRNA pathway, RdRs and DCL proteins, function in biotic and abiotic stress responses [42-45]. Silencing of *N. attenuata RdR1*, *DCL3*, and *DCL4* results in impaired defense responses against *M. sexta* herbivory [42,44,45]. Silencing either *NaRdR1* or *NaDCL4* impairs jasmonic acid (JA) accumulation, and co-silencing *NaDCL3* and *NaDCL4* reduces JA levels, indicating that RdR1/DCL4-mediated smRNAs are critical regulators of responses to insect herbivory.

To deepen our understanding of the roles that smRNAs play in plant-insect interactions, we identified primary miRNA (*MIR*) transcripts and *TAS* transcripts encoding tasiRNAs in a transcriptome database of *N. attenuata*[46], and computationally analyzed secondary stem-and-loop structures of *MIR* transcripts. To understand the role of jasmonates in regulating miRNAs, we examined miRNA accumulation in jasmonate-deficient *allene oxide cyclase* (*AOC*) RNAi lines. The AOC protein provides a precursor for JA biosynthesis [47]. Expression analysis of miRNAs and tasiRNAs with their putative target genes provides evidence for a key role of plant smRNAs in the response to herbivory.

## Results and discussion

### Identification of conserved miRNAs and their precursors in *N. attenuata*

**Figure 1:**
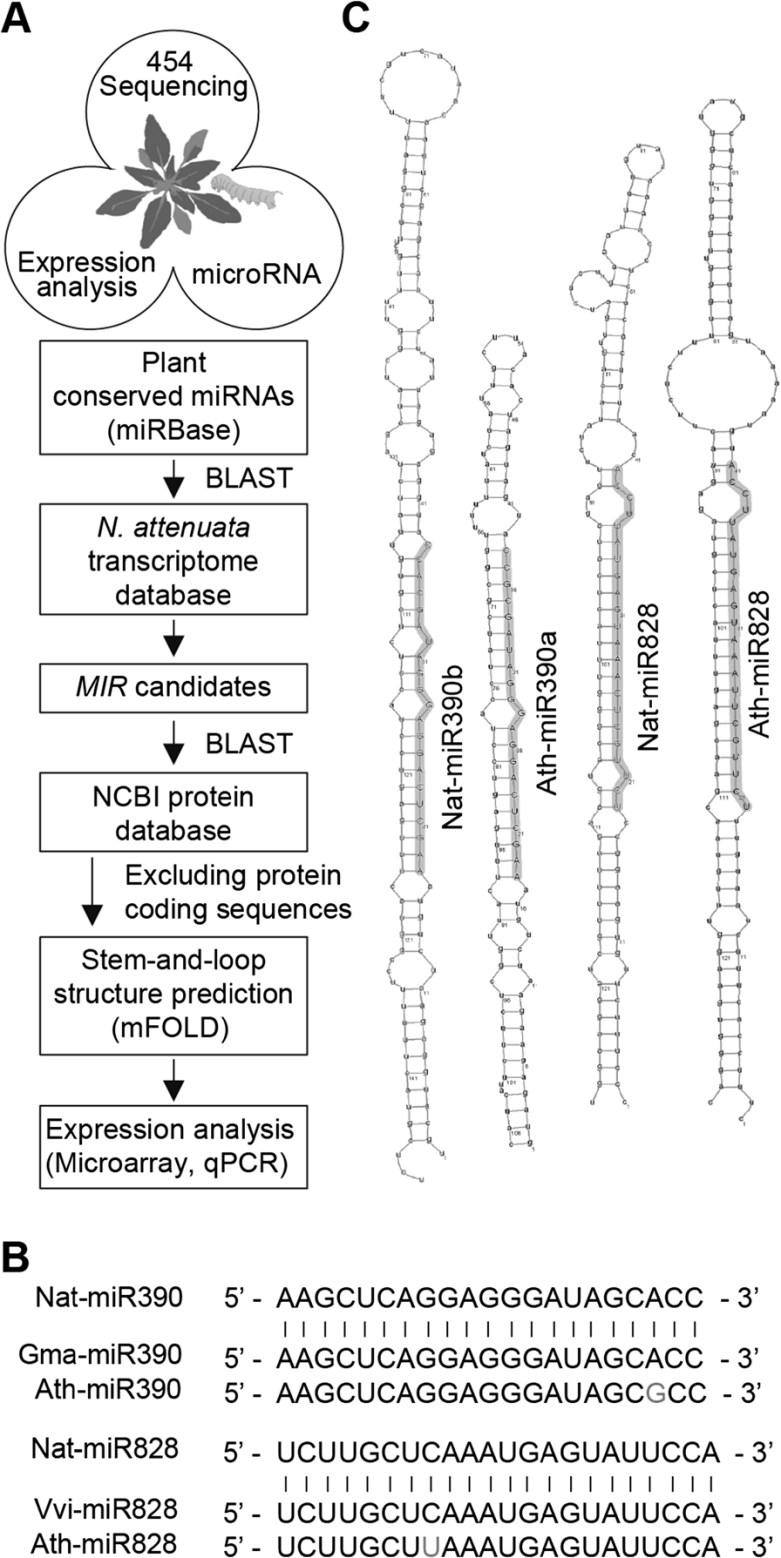
**Identification and prediction of miRNAs in *N. attenuata.*** (**A**) A workflow depicting miRNA identification in *N. attenuata*. *MIR*, primary miRNA transcript. (**B**) Examples of conserved *N. attenuata* miRNAs with orthologs in plant species. Ath, *Arabidopsis thaliana*; Gma, *Glycine max*; Nat, *Nicotiana attenuata*; Vvi, *Vitis vinifera*. (**C**) Stem-and-loop structures of Nat-miR390 and Nat-miR828 precursors. Hairpin structures are compared to *Arabidopsis* miRNA orthologs based on their miRNAs sequence similarity. The miRNAs are highlighted in structures.

To identify conserved miRNAs in *N. attenuata*, we used a 454-transcriptome database of *N. attenuata* to conduct a BLAST search against conserved plant miRNAs in the miRBase ( http://www.mirbase.org) (Figures 1A and 1B). This search identified 59 potential miRNAs distributed in 36 families (Table 1). We used the BLASTX algorithm against the NCBI protein database to check that the putative primary transcripts of miRNAs were non-coding. Web-based mFOLD software ( http://mfold.rna.albany.edu/) was used to predict secondary stem-and-loop structures. Of the identified miRNA-precursors, 52 had stem-and-loop structures (Figure 1C and Additional file 1), which were created with minimum free energies (MFE) ranging from ΔG = −97.5 kcal mol^-1^ to −33.3 kcal mol^-1^ (Table 1) with an average MFE of −62.1 kcal mol^-1^. This average MFE is comparable to that found in *A. thaliana* (−59.5 kcal mol^-1^), higher than in the red alga *Porphyra yezoensis* (−41.7 kcal mol^-1^) and lower than in the monocots rice (−71.0 kcal mol^-1^) and wheat (−72.4 kcal mol^-1^) [22,48]. Only seven predicted miRNA-precursors transcripts did not form stem-and-loop structures or were not stable (Table 1). We identified several *N. attenuata* (Nat) miRNA families (Nat-miR403, Nat-miR478, Nat-miR482, Nat-miR1128, Nat-miR1133, Nat-miR1446, Nat-miR1863, Nat-miR2911, and Nat-miR5281) which were not reported in *N. tabaccum*[28,49]. Among these, Nat-miR478, Nat-miR482, Nat-miR1128, Nat-miR1133, Nat-miR1446, Nat-miR1863, and Nat-miR5281are absent in *A. thaliana* but are close homologues to those in other plant species (Table 1).

**Table 1:** **Identification and prediction of miRNAs in *N. attenuata***

**Nat-MIR family members**	**MFE**	**miRNA**	**Length (nt)**	**GC content**	**Hit in miRBase**	**E-value**
				**(%)**		
Nat-miR156a	−55.1	ugacagaagagagugagcaca	21	47.6	bna-miR156	0.001
Nat-miR156b	−76.5	ugacagaagagagugagcaca	21	47.6	bna-miR156	0.001
Nat-miR157	−43.9	uugacagaagauagagagcac	21	42.9	ath-miR157	0.001
Nat-miR159a	−62.3	uuuggauugaagggagcucua	21	42.9	ath-miR159	0.001
Nat-miR159b	−90.6	uuuggauugaagggagcucua	21	42.9	ath-miR159	0.001
Nat-miR159c	−104.3	uuuggauugaagggagcucua	21	42.9	ath-miR159	0.001
Nat-miR160a	−61.6	ugccuggcucccuguaugcca	21	61.9	ath-miR160	0.001
Nat-miR160b	−63.3	ugccuggcucccuguaugcca	21	61.9	ath-miR160	0.001
Nat-miR162	−43.9	uggaggcagcgguucaucgauc	22	54.5	csi-miR162	0.0001
Nat-miR164	−74.2	uggagaagcagggcacgugca	21	61.9	ath-miR164	0.001
Nat-miR166a	−59.8	ucggaccaggcuucauucccc	21	61.9	ath-miR166	0.001
Nat-miR166b	−51	ucggaccaggcuucauucccc	21	61.9	ath-miR166	0.001
Nat-miR166c	−71.8	ucggaccaggcuucauucccc	21	61.9	ath-miR166	0.001
Nat-miR167a	−42	ugaagcugccagcaugaucua	21	47.6	ath-miR167	0.001
Nat-miR167b	−47	ugaagcugccagcaugaucua	21	47.6	ath-miR167	0.001
Nat-miR168	−78.3	cccgccuugcaucaacugaau	21	52.4	aly-miR168	0.001
Nat-miR169a	−86.1	cagccaaggaugacuugccga	21	57.1	ath-miR169	0.001
Nat-miR169b	−70.5	uagccaaggaugacuugccugc	22	50.0	bna-miR169	0.001
Nat-miR171a	−70.1	ugauugagccgcgucaauauc	21	47.1	vvi-miR171	0.001
Nat-miR171b	−61.4	ugauugagccgcgucaauauc	21	47.1	vvi-miR171	0.001
Nat-miR171c	−44.9	ugauugagccgcgucaauauc	21	47.1	vvi-miR171	0.001
Nat-miR171d	−60.4	ugauugagccgcgucaauauc	21	47.1	vvi-miR171	0.001
Nat-miR172a	−53.4	ugagaaucuugaugaugcugcau	23	39.1	vvi-miR172	0.001
Nat-miR172b	−49.5	ugagaaucuugaugaugcugcau	23	39.1	vvi-miR172	0.001
Nat-miR172c	−61.7	ugagaaucuugaugaugcugcau	23	39.1	vvi-miR172	0.001
Nat-miR172d	−35.2	ugagaaucuugaugaugcugcau	23	39.1	vvi-miR172	0.001
Nat-miR319a	−97.5	uuggacugaagggagcucccu	21	57.1	ath-miR319	0.001
Nat-miR319b	−89.7	uuggacugaagggagcucccu	21	57.1	ath-miR319	0.001
Nat-miR319c	−95.4	uuggacugaagggagcucccu	21	57.1	ath-miR319	0.001
Nat-miR390a	−67.6	aagcucaggagggauagcacc	21	57.1	gma-miR390	0.001
Nat-miR390b	−58.1	aagcucaggagggauagcacc	21	57.1	gma-miR390	0.001
Nat-miR393a	−41.7	uccaaagggaucgcauugaucc	21	45.5	ath-miR393	0.0004
Nat-miR393b	−54.4	uccaaagggaucgcauugaucc	21	45.5	ath-miR393	0.0004
Nat-miR394a	−41.7	uuggcauucuguccaccuccau	22	50.0	vvi-miR394	0.0004
Nat-miR394b	−82.3	uuggcauucuguccaccuccau	22	50.0	vvi-miR394	0.0004
Nat-miR396	−56.4	uuccacagcuuucuugaacug	22	42.9	ath-miR396	0.001
Nat-miR397	−49.9	ucauugagugcagcguugaug	22	47.6	ath-miR397	0.001
Nat-miR398	−69.1	uguguucucaggucaccccuu	21	52.4	ath-miR398	0.001
Nat-miR399	-	ugccaaagaagauuugccccgu	21	52.4	ptc-miR399	0.001
Nat-miR403	−38.2	uuagauucacgcacaaacucg	21	42.9	ath-miR403	0.001
Nat-miR408	−47.6	augcacugccucuucccuggc	21	61.9	ath-miR408	0.001
Nat-miR413	-	cuaguuucucuuguucugcuu	21	38.1	ath-miR413	0.015
Nat-miR414	-	uccucuucaucaucaucuuc	21	40.0	ath-miR414	0.074
Nat-miR477	−54.8	acucucccucaagggcuucug	21	57.1	aqc-miR477	0.001
Nat-miR478	−33.3	ugacaugucuuauauuuuuag	20	23.8	ptc-miR478	0.005
Nat-miR482	−53.1	uuuccaauuccacccauuccua	21	40.9	sly-miR482	0.0004
Nat-miR828	−46.7	ucuugcucaaaugaguauucca	21	36.4	vvi-miR828	0.0004
Nat-miR845a	-	ugcucugauaccaaauugaug	22	38.1	ath-miR845	0.003
Nat-miR845b	-	uggcucugauaccaauugau	22	40.0	vvi-miR845	0.004
Nat-miR1128	−85.4	uacuacucccuccguuucaa	20	45.0	ssp-miR1128	0.081
Nat-miR1133	−88.1	cauauacucccuccgucccugaaa	21	50.0	tae-miR1133	0.017
Nat-miR1446	−48.7	uucugaacucucucccucaa	20	45.0	ptc-miR1446	0.003
Nat-miR1863a	-	gcucugauaccauguuaacu	24	40.0	osa-miR1863b	0.008
Nat-miR1863b	-	gacucugauaccauguuaaaauag	20	28.0	osa-miR1863	0.02
Nat-miR1919	−87.1	aggcgagtcatctgtgacagg	21	57.1	sly-miR1919	0.029
Nat-miR2911	−67.3	ggccgggggacggacuggga	20	80.0	peu-miR2911	0.014
Nat-miR5281a	−51.9	cauauaaauugaaacggagggag	23	39.1	mtr-miR5281b	0.13
Nat-miR5281b	−69.9	cauauaaauugaaacggagggag	23	39.1	mtr-miR5281b	0.13
Nat-miR5281c	−34.2	cauauaaauugaaacggagggag	23	39.1	mtr-miR5281b	0.13

Next, we designed probes to detect *N. attenuata* miRNAs on RNA blots ( Additional file 2). We performed northern blot hybridization using 40 μg of total RNA extracted from rosette leaves to detect selected miRNAs. Accumulation of miRNAs varied ( Additional file 3). Accumulation of Nat-miR159, Nat-miR171, Nat-miR172, and Nat-miR319 was high compared to Nat-miR157, Nat-miR393, Nat-miR396, and Nat-miR828 in leaves from rosette-stage plants. For further analyses of precursor and mature miRNA abundance, we used real-time quantitative PCR (qPCR) with specific primer sets ( Additional file 4 and 5).

### Identification of conserved tasiRNAs in *N. attenuata*

**Figure 2:**
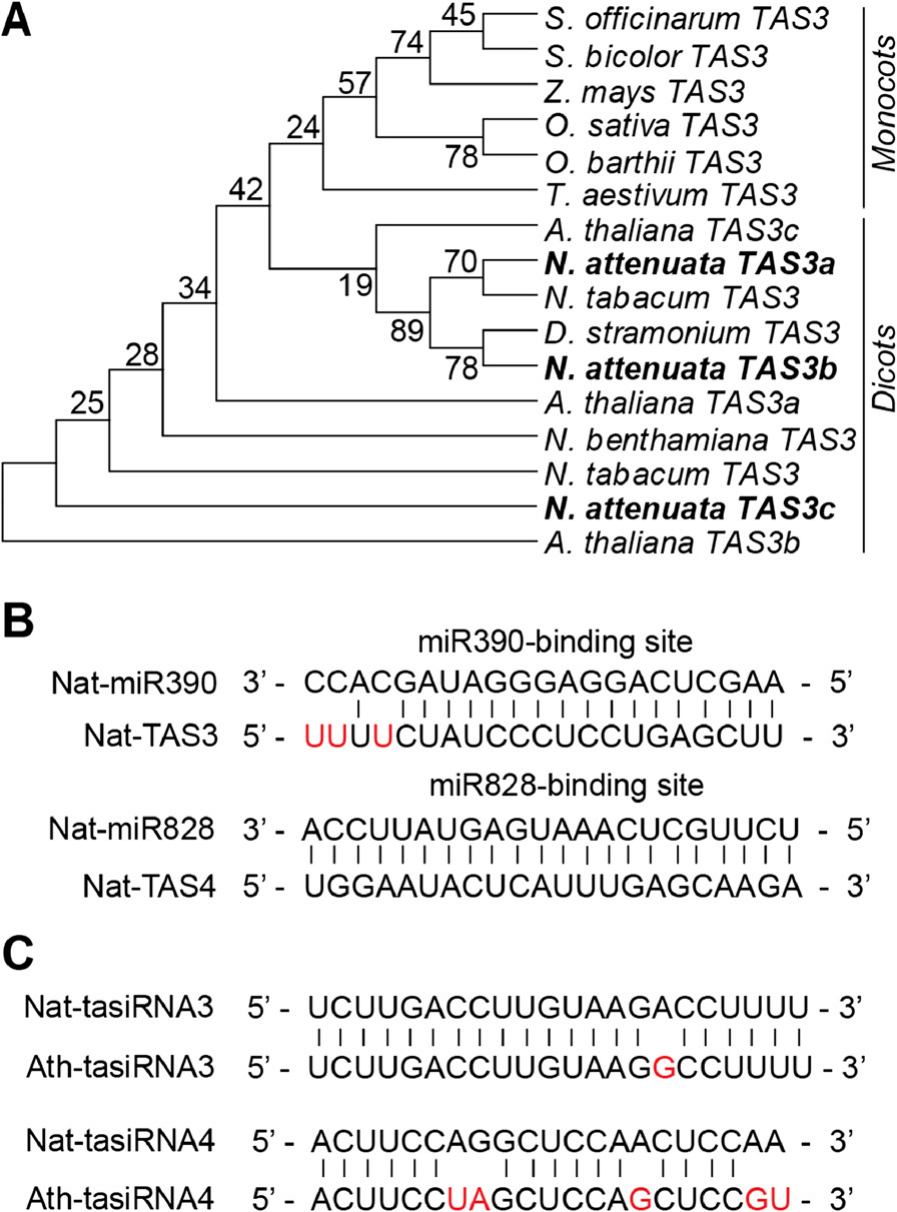
**Identification of miRNA-regulated tasiRNAs in *N. attenuata.*** (**A**) Phylogenetic analysis of three *TAS3* transcripts in *N. attenuata*. Nucleotide sequences of three *TAS3* transcripts were aligned with *TAS3* orthologs of monocotyledonous and dicotyledonous plants. Distance values were calculated using the neighbor-joining method with 1000 bootstrap replicates. (**B**) Binding site of Nat-miR390 and Nat-miR828 in *TAS3* and *TAS4* transcript, respectively. (**C**) Conserved tasiRNAs in *N*. *attenuata* and *A*. *thaliana*.

Four families of endogenous tasiRNAs (*TAS1*, *TAS2*, *TAS3*, and *TAS4*) identified in *A. thaliana* are regulated by miRNAs [9,50]. We found three *TAS3* transcripts and one *TAS4* transcript in *N. attenuata* (Figure 2), and constructed a phylogenetic tree with their homologs from different plant species to examine the evolutionary relationships of *TAS3* expressed in dicotyledonous and monocotyledonous plant species [50,51]. Not surprisingly, *NaTAS3* members were grouped amongst members of the dicotyledonous plant species (Figure 2A).

Because *TAS3* and *TAS4* transcripts contain the binding sites of miR390 and miR828, respectively [9,11,52], we blasted Nat-miR390 and Nat-miR828 against the *TAS* transcripts (Figure 2B), and compared the *N. attenuata* tasiRNAs with those in *A. thaliana* (Figure 2C). Sequence analysis indicated that binding sites of miRNA and tasiRNA sequences are highly conserved in *N. attenuata* and *A. thaliana*.

### Wound- and OS-inducible miRNAs

**Figure 3:**
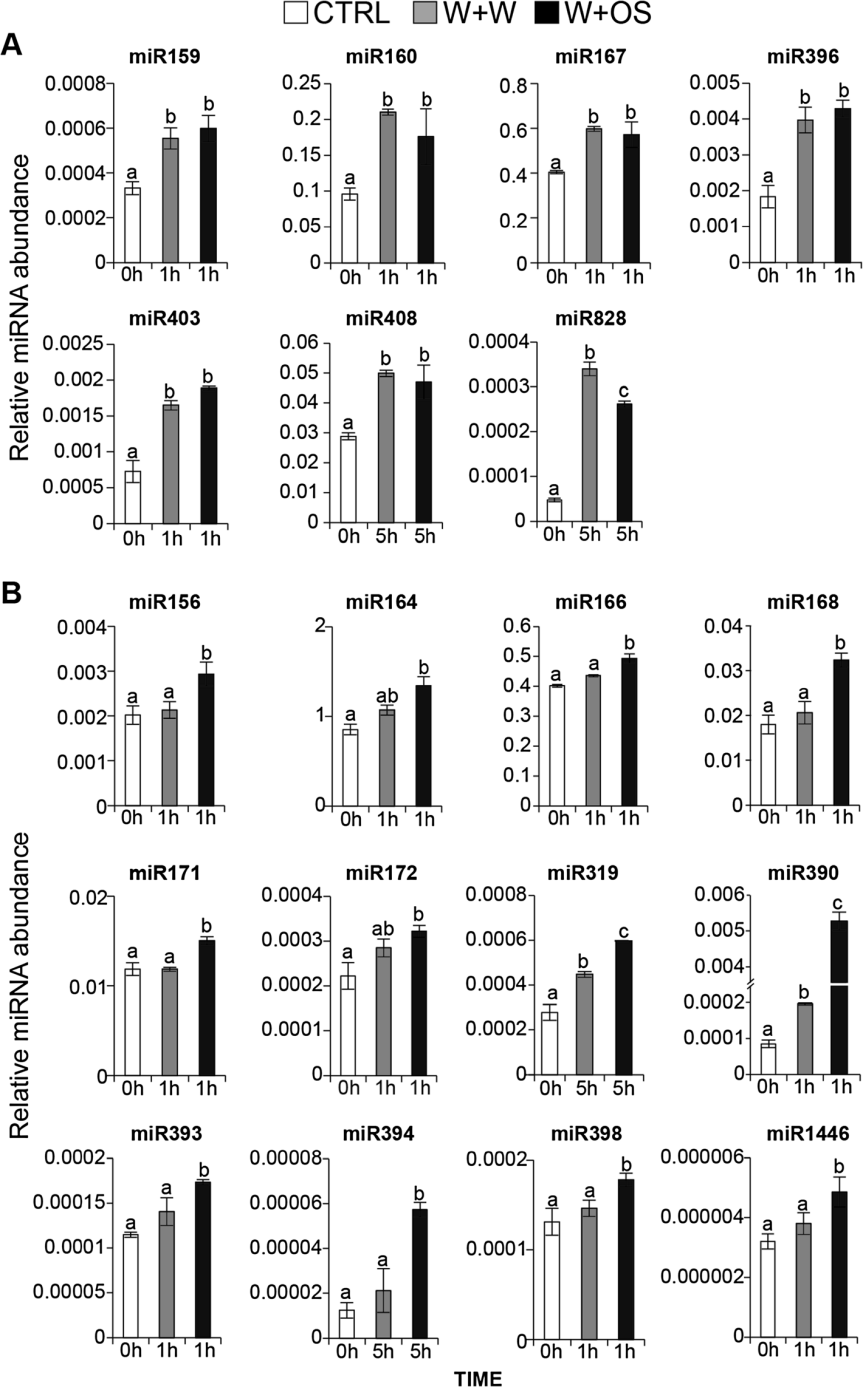
**Wound- and OS-inducible miRNAs.** (**A**) Wound-inducible miRNAs. (**B**) OS-inducible miRNAs. Effects of W+W and W+OS on relative transcript abundance of *N. attenuata* miRNAs. For each sample, one leaf on the rosette of a 32-day-old WT plant was left untreated. (control, CTRL) or treated with wounding plus water (W+W) or wounding plus oral secretions of the larvae of the specialist *M. sexta* (W+OS) and harvested 1 h or 5 h post treatment. Shown are mean (± SE) levels of three replicates. Letters indicate significant differences (*P* < 0.05) in Fisher’s PLSD tests following an ANOVA.

**Figure 4:**
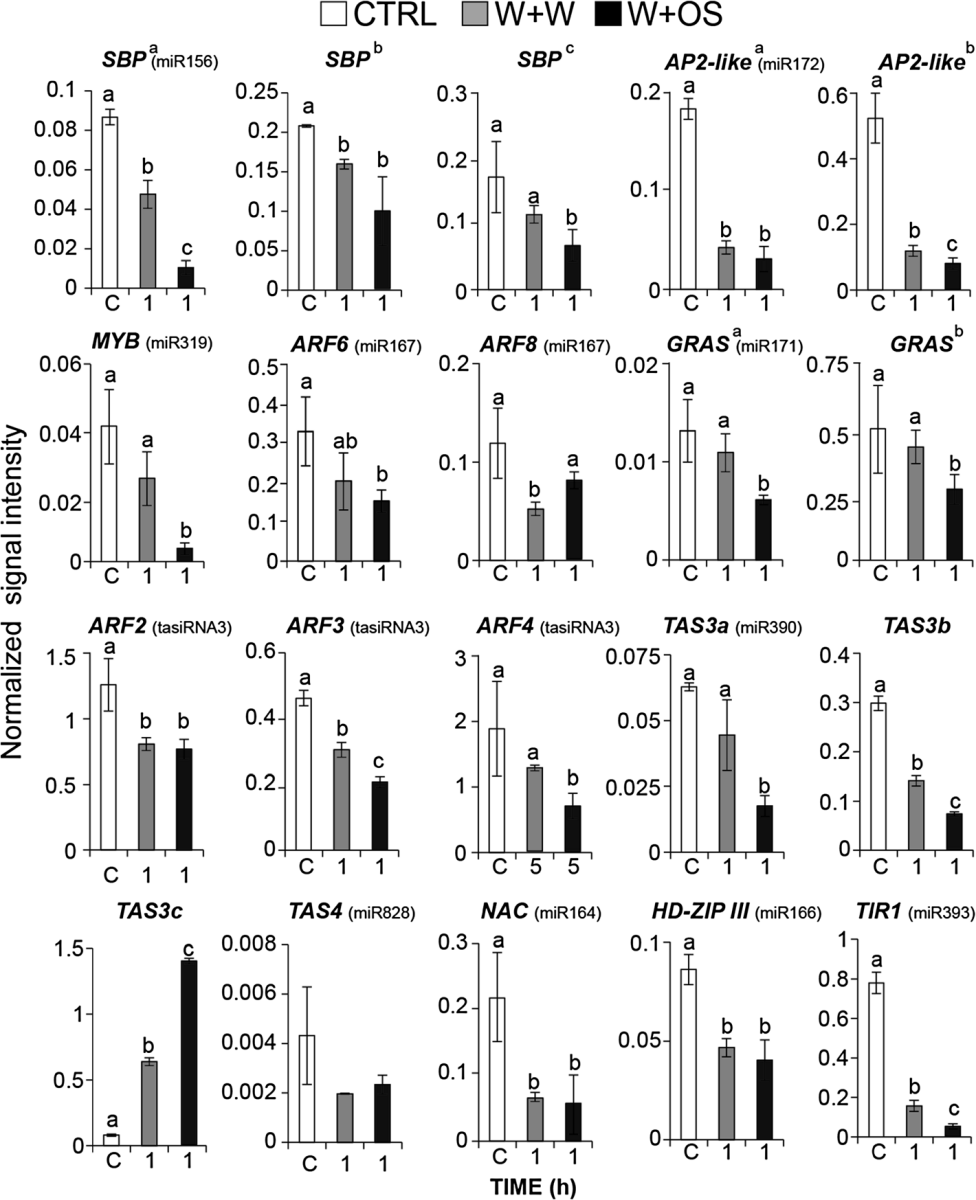
**Target genes expression of miRNAs and tasiRNAs.** Mean (± SE) levels of normalized transcript abundance of miRNAs and tasiRNAs target genes after W+W and W+OS treatments. Untreated plants served as control (CTRL, C). Signal intensities in microarray data was normalized using the 75th percentile value and log2-transformation. Letters indicate significant differences (*P* < 0.05) in Fisher’s PLSD test following an ANOVA. For identification of the target genes, see Additional file 9.

Next, we examined the abundance of miRNAs and their target genes in leaves of rosette-stage WT plants changed after wounding and the application of diluted *M. sexta* OS (W+OS) or water to wounds (W+W, as a control for W+OS) versus no treatment (control) (Figures 3 and 4). W+OS treatment faithfully mimics the majority of responses elicited by *M. sexta* feeding [36,38], but has the distinct advantage of allowing the time of elicitation to be precisely controlled (as opposed to the sporadic nature of *M. sexta* larval feeding behavior) and hence greatly increases the reproducibility of transcriptional analyses.

The abundance of mature Nat-miR159, Nat-miR160, Nat-miR167, Nat-miR396, Nat-miR403, Nat-miR408, and Nat-miR828 increased after both W+W and W+OS treatments, abundances of which did not differ (Figure 3A). We categorized these miRNAs wound-inducible miRNAs. Twelve conserved miRNA families (Nat-miR156, Nat-miR164, Nat-miR166, Nat-miR168, Nat-miR171, Nat-miR172, Nat-miR319, Nat-miR390, Nat-miR393, Nat-miR394, Nat-miR398, and Nat-miR1446) were significantly increased in W+OS treated leaves compared to control and W+W treated leaves (Figure 3B) and these were classified as OS-inducible miRNAs.

It is well-established that miRNAs and tasiRNAs bind to their target mRNAs by perfect or imperfect complementarity [5]. Such complementarity permits the identification of miRNA targets in databases. We blasted miRNA sequences against an in-house transriptomic database using BLASTN algorithms with default parameters allowing 1–4 mistmatches. We identified 111 potential targets, inluding targets of tasiRNAs ( Additional file 6).

Ath-miR164 is known to negatively regulate ORE1/NAC2 transcription factors which are involved in age-dependent senescence [53] and mutations in ORE1/NAC2 delays chlorophyll loss in old leaves of *A. thaliana*[53]. We found a NAC transcription factor containing a Nat-miR164 binding site in *N. attenuata* (Figure 4). The NAC transcripts were significantly reduced in W+W and W+OS elicited leaves (Figure 4), which correlated with elevated Nat-miR164 levels after OS-elicitation (Figure 3B). This result may explain how *M. sexta* attack delays senescence in *N. attenuata*[54].

In addition, *ARF6* and *ARF8* were putative targets of wound-inducible Nat-miR167, and their transcripts decreased concurrently with the accumulation of Nat-miR167 (Figure 4). Potential targets of OS-inducible Nat-miR171, GRAS domain transcription factors, were down-regulated in OS-elicited plants (Figure 4). Transcripts of several OS-inducible Nat-miR156 targets annotated as *SQUAMOSA* promoter binding proteins (SBPs) were significantly down-regulated after W+OS treatment. Abundance of Nat-miR172 increased significantly in W+OS treated leaves (Figure 3B) and consistently, transcripts of its putative targets, AP2-like proteins, were significantly down-regulated in W+W and W+OS treated leaves (Figure 4).

**Figure 5:**
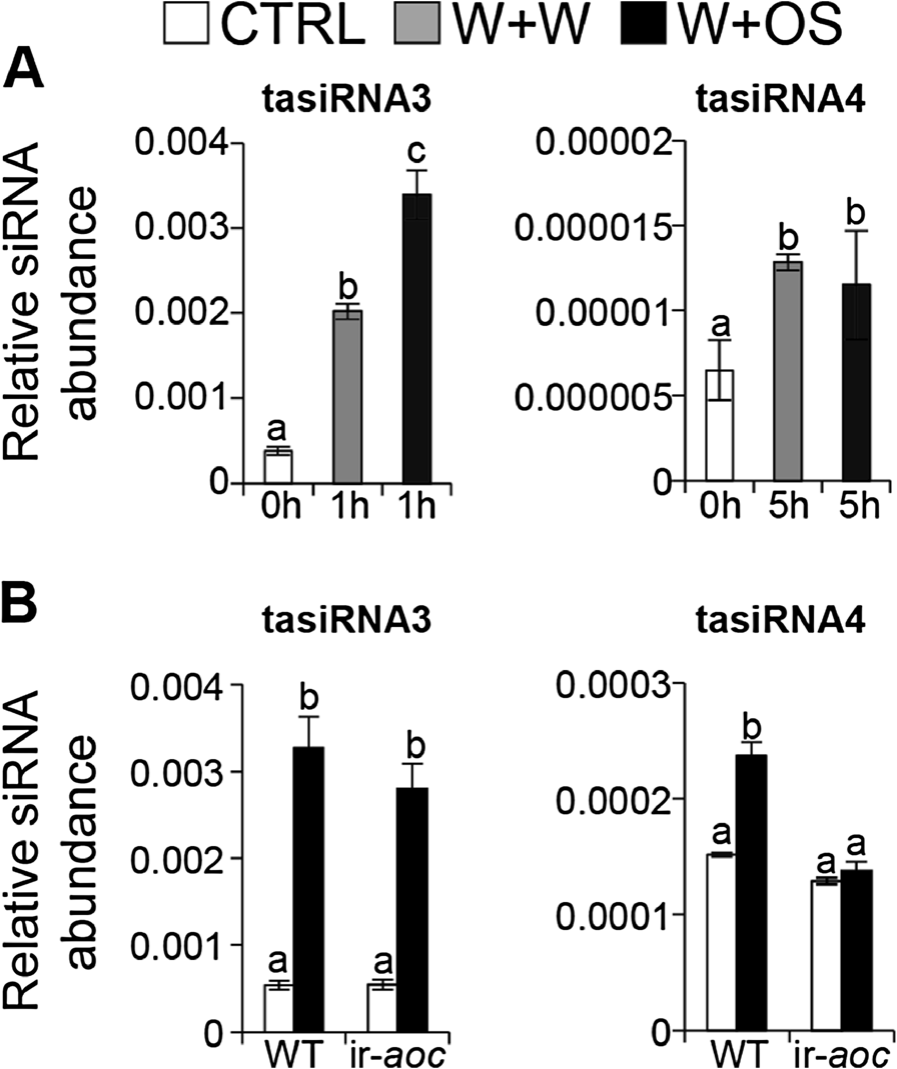
**Accumulation of tasiRNAs in response to OS-elicitation.** (**A**) Abundance of tasiRNA3, which is a target of the OS-inducible Nat-miR390, significantly increased in OS-elicited plant compared to control (CTRL) and W+W treatment. Abundance of tasiRNA4, which is regulated by the wound-inducible Nat-miR828, was significantly increased by wounding. (**B**) Accumulation of JA-independent tasiRNA3 and JA-dependent tasiRNA4 at 1 h and 5 h after OS-elicitation, respectively. For each sample, one leaf on the rosette of a 32-day-old plant was treated with wounding plus oral secretions of the larvae of the specialist herbivore *M. sexta* (W+OS) and harvested 1 h or 5 h post treatment. Untreated plants served as control. Shown are mean (± SE) levels of three replicates. Letters indicate significant differences (*P* < 0.05) in Fisher’s PLSD test following an ANOVA.

The most significant change in miRNA transcripts after W+OS treatment was for Nat-miR390, which showed a 75-fold increase compared to control leaves and a 30-fold increase compared to W+W treated leaves (Figure 3B). Ath-miR390 cleaves *TAS* transcripts, resulting in production of tasiRNA3 mediated by the RdR6/DCL4 complex [11,52]. TasiRNA3 controls the transcription of auxin response factors (ARFs) 2, 3, and 4, which regulate leaf morphology and lateral root growth in *A. thaliana*[11,16,52]. Overexpression of *TAS3* leads to an increased number of lateral roots, and the knock-out mutant shows impaired lateral root growth [11]. Abundance of mature Nat-tasiRNA3 in W+OS treated leaves was increased (Figure 5A) and Nat-tasiRNA3 was regulated in a JA-independent manner (Figure 5B). The targets of tasiRNA3, transcripts homologous to *A. thaliana ARF2*, *3* and *4* were significantly reduced after W+OS elicitation (Figure 4), suggesting that Nat-miR390 could affect the architecture of roots and thereby regulate the production of nicotine, which is synthesized in the roots, or promote tolerance of herbivory by increasing mineral uptake or sugar storage in roots. Our previous study shows that silencing *NaDCL4*, which in turn reduces the accumulation of tasiRNA3, dramatically impairs root growth and nicotine accumulation in *N. attenuata*[45]. We validated the microarray data by qPCR analysis ( Additional file 7), and the result showed similar expression patterns ( Additional file 8).

### JA-dependent miRNAs

The key role of jasmonates in mediating responses to herbivory is well established and it was not surprising to find that the levels of jasmonates dramatically increased in W+OS treated leaves compared to W+W treatment ( Additional file 9) [37]. Silencing of *NaAOC* reduces JA accumulation in W+OS treated leaves by 90-100% ( Additional file 9) and is known to silence the production of JA-mediated defenses [47]. OS-elicitation of this genotype allowed us to understand which miRNAs are regulated by jasmonates.

**Figure 6:**
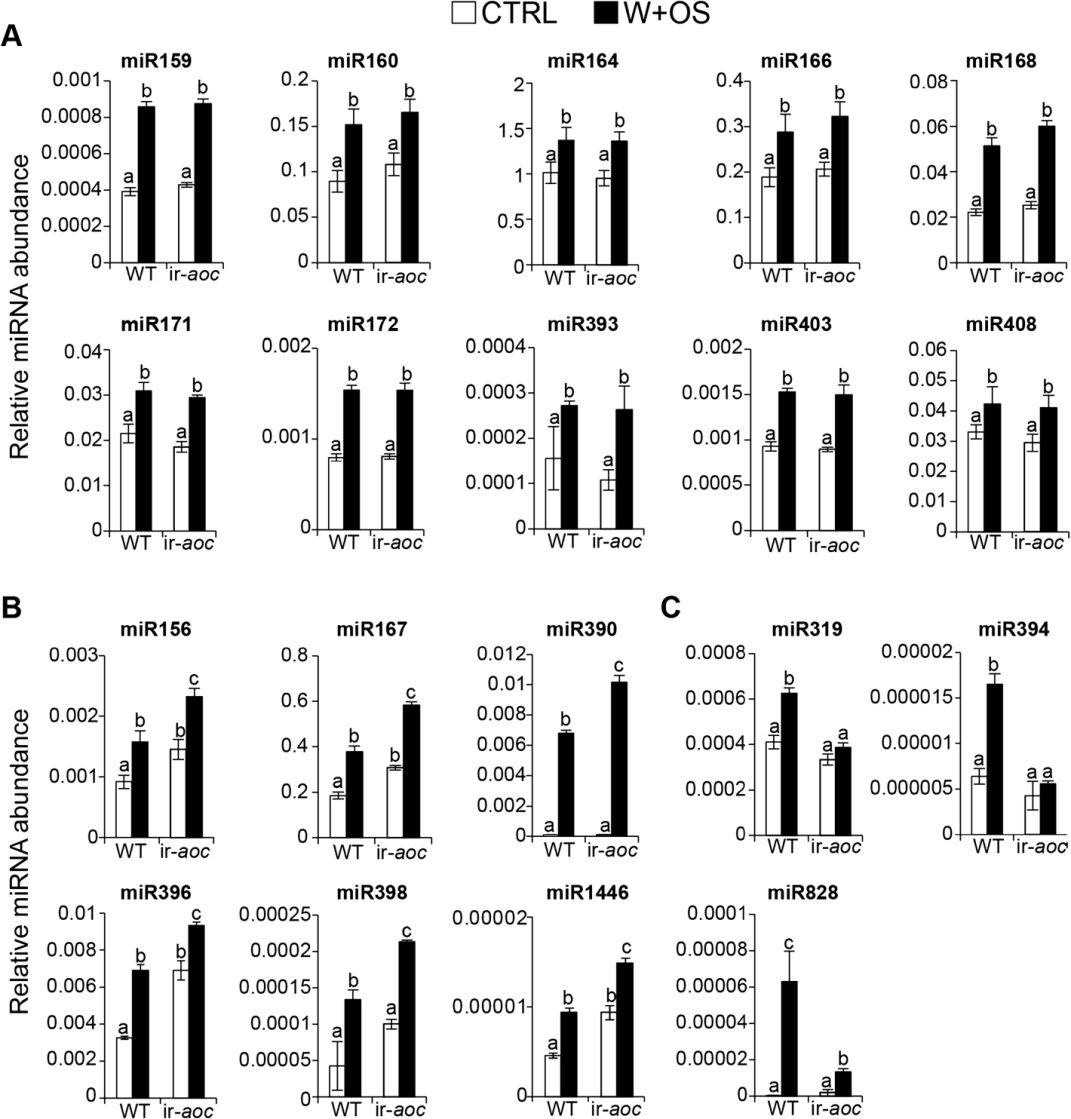
**JA-independent and dependent miRNAs accumulation in response to OS-elicitation.** (**A**) W+OS inducible and JA-independent miRNAs. (**B**) W+OS inducible miRNAs accumulated more in ir-*aoc* plant. (**C**) W+OS inducible and JA-dependent miRNAs. Shown are mean (± SE) levels of miRNAs in control and elicited leaves at 1 h or 5 h after W+OS treatment in wild-type (WT) and ir-*aoc* plants. For each sample, one leaf on the rosette of a 32-day-old WT and ir-*aoc* plant was left untreated (control, CTRL) or treated with wounding plus oral secretions of the larvae of the specialist herbivore *M. sexta* (W+OS) and harvested 1 h or 5 h post elicitation. Letters indicate significant differences (*P* < 0.05) in Fisher’s PLSD tests following an ANOVA.

Abundance of 10 miRNAs (Nat-miR159, Nat-miR160, Nat-miR164, Nat-miR166, Nat-miR168, Nat-miR171, Nat-miR172, Nat-miR393, Nat-miR403, and Nat-miR408) increased after W+W and W+OS treatments, but did not differ between WT and ir-*aoc* plants (Figure 6A). We considered these miRNAs as JA-independent miRNAs. The abundance of Nat-miR156, Nat-miR167, Nat-miR390, Nat-miR396, Nat-miR398, and Nat-miR1446 were significantly higher in both control and W+OS elicited leaves of ir*-aoc* than of WT plants (Figure 6B), indicating that jasmonates or JA-signaling negatively influences levels of these miRNAs, regardless of treatment. Nat-miR390 abundance was higher in ir-*aoc* only after W+OS treatment (Figure 6B). While abundance of Nat-miR319 and Nat-miR394 were induced by W+OS treatment in WT (Figure 3B), they were not induced by W+OS treatment in ir-*aoc* plants (Figure 6C). We considered these as OS-inducible JA-dependent miRNAs. Wound-inducible Nat-miR828 was induced in both W+W and W+OS treatments in WT (Figure 3A), but their levels were not dramatically induced in elicited JA-deficient ir-*aoc* plant compared to WT (Figure 6C).

One main target of miR319 in *A. thaliana* is TCP (TEOSINTEBRANCHED/CYCLOIDEA/PCF) transcription factors, which positively regulates jasmonate biosynthesis [18]. The TCP4 protein can bind a promoter of *LIPOXYGENASE2* (*LOX2*) in *A. thaliana*[18]. We do not yet know the orthologs of *A. thaliana* TCPs in *N. attenuata*, but the timing of Nat-miR319 induction was similar with that of W+OS elicited JA accumulation and the induction of *NaLOX3*, which is the functional homolog of *A. thaliana LOX2*[40,55]. W+OS treatment amplifies the wound-induced JA accumulation ( Additional file 9) [36,38] and does the same for Nat-miR319 (Figure 3B). This suggests that Nat-miR319 could play a role in the fine-tuning regulation of jasmonate biosynthesis [18] during herbivory.

Nat-miR828 and Nat-tasiRNA4 were increased by W+W and W+OS treatments in WT (Figure 3A), but not in ir-*aoc* plants (Figures 5 and 6C). Levels of Nat-miR828 dramatically decreased in ir-*aoc* plants compared to WT and its primary transcript as well (Figure 6C and Additional file 10). One target of miR828 is a *TAS4* transcript, which encodes tasiRNA4 processed by RdR6/DCL4 proteins [9,56]. TasiRNA4 targets several MYB transcription factors which regulate phenylpropanoid biosynthesis [9,56]. Several phenylpropanoid defense metabolites increase in *N. attenuata* during herbivory and silencing of *DCL4* alters the accumulation of dicaffeoyl spermidine and caffeoylputrescine in W+OS-elicited leaves [45]. A jasmonate-inducible MYB transcription factor, NaMYB8, is involved in plant defense against herbivory and phenylpropanoid biosynthesis [57,58]. Although we were not able to find a MYB transcription factor containing a Nat-tasiRNA4 binding site in our current cDNA library, sequence conservation of miR828 and tasiRNA4 in *A. thaliana* and *N. attenuata* will guide the identification of targets which regulate secondary metabolite production.

### Temporal effect of single elicitation on miRNAs and their targets accumulation

To examine the kinetics of miRNAs in response to insect herbivory, we measured the accumulation of miRNAs and their targets at 1 h and 5 h after treatments. Most OS-induced miRNAs increased in abundance within 1 h after OS-elicitation and continue to increase until 5 h, except miR166 and miR390 ( Additional file 11). The miRNA390 increased at 1 h but decreased again by 5 h post-elicitation ( Additional file 11). OS-inducible miRNAs were also induced by wounding but the increase after wounding was less (miR164, miR166, miR172, miR319, and miR398) or slower (miR168, miR390, and miR393) than that after OS-elicitation. Interestingly, most of the miRNA and tasiRNA target genes were reduced at 1 h after OS-elicitation but return to control levels (SBPa, ARF2, ARF3, TAS3a, TAS3b, HD-ZIPIII, TIR1) or even increased to levels greater than prior to elicitation (MYB, ARF6, ARF8), suggesting a complex relationship between the regulator mature miRNAs and their target genes.

### Comparison between primary and mature miRNA transcript accumulation

We checked the accumulation of *MIR* transcripts using previously reported microarray data [46] ( Additional file 12) and confirmed these responses by qPCR ( Additional file 10). Accumulation of *MIR156*, *MIR159*, *MIR164*, *MIR166*, *MIR167*, *MIR168*, *MIR172*, *MIR393*, *MIR396*, *MIR398*, *MIR403*, and *MIR1446* was not correlated to the abundance of their mature miRNAs. Changes in transcript levels of *MIR160*, *MIR171*, *MIR319*, *MIR390*, *MIR408*, and *MIR828* reflected the transcript accumulation of their mature miRNAs. Generally, a weak relationship between *MIR* and mature miRNA accumulation has been reported [59]. Our data also suggests that the processing of *MIR* transcripts is more important than the transcription of *MIR* genes in herbivory-induced miRNA regulation.

## Conclusion

**Figure 7:**
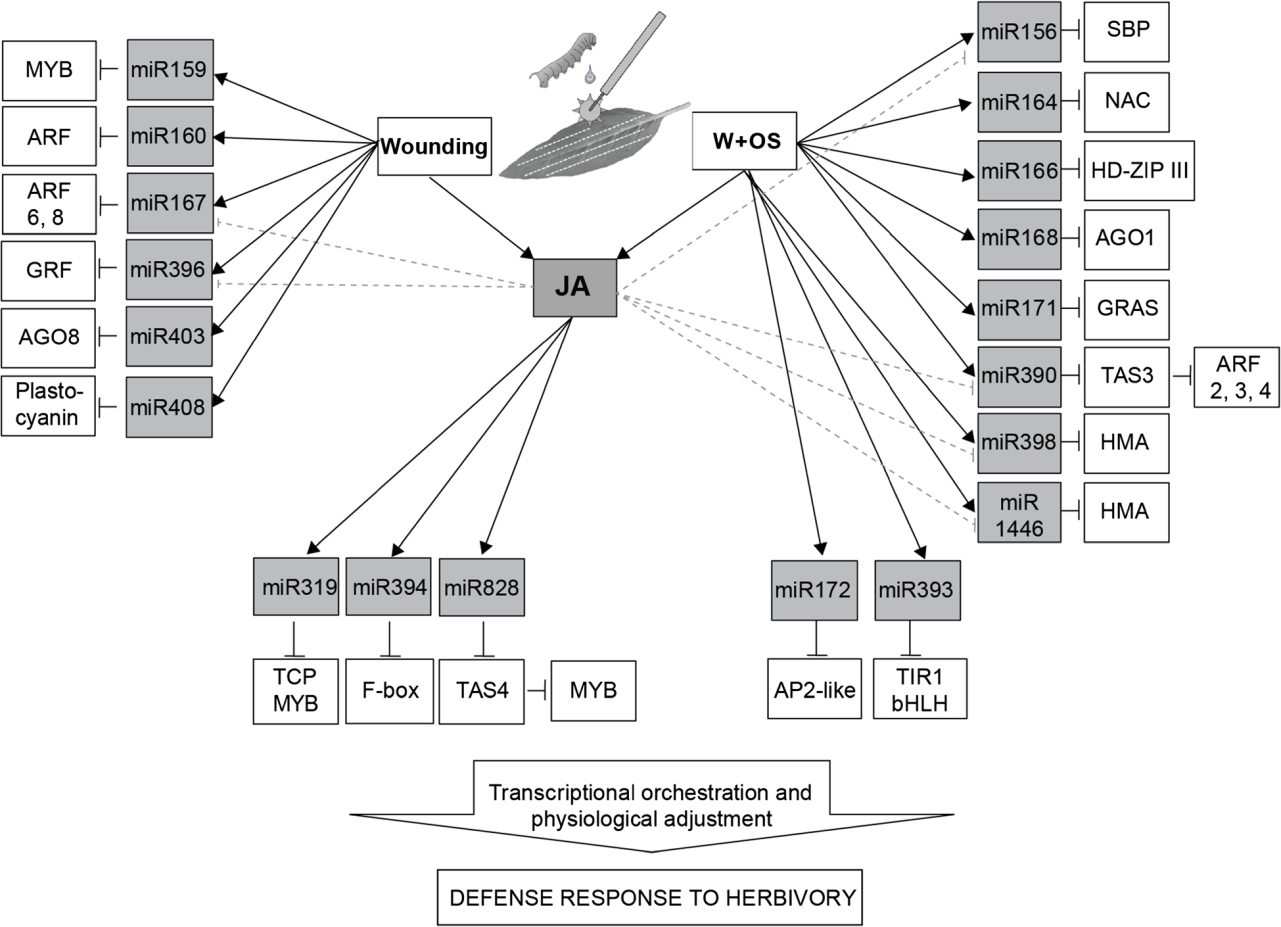
**Summary of herbivory-responsive miRNAs and tasiRNAs in *N. attenuata***.

In order to build a database of plant miRNAs functionally involved in plant-insect interactions, we investigated the accumulations of miRNAs and their targets in *N. attenuata* after OS-elicitation. We classified W+OS-induced miRNAs and tasiRNAs into four groups: JA-dependent or –independent wound-inducible and OS-inducible miRNAs or tasiRNAs (Figure 7). Herbivore-attacked *N. attenuata* plants induce defense metabolites and tune their physiology to tolerate insect attack. This study shows that W+OS elicitation, a rigorous means of mimicking herbivore attack, rapidly changed the expression of miRNAs involved in flowering time, root morphology, senescence, hormone regulation, and metabolite synthesis. In future work, we will experimentally characterize the function of these W+OS regulated JA-dependent and JA-independent miRNAs and their targets.

## Methods

### Plant material and growth conditions

WT and JA-deficient ir-*aoc* RNAi lines of the 30th inbred generation of the *N. attenuata* (Torrey ex. Watson) (originally collected in Southwestern Utah, USA) were used for the experiments. Seeds were germinated on MS basal medium supplemented with vitamin B5 (GB5, Duchefa, http://www.duchefa.com). For treatment, the leaf at the +1 node of the rosette (youngest fully-expanded leaf) was wounded with a fabric pattern wheel, and either 20 uL MilliQ water (W+W) or 20 μL *M. sexta* OS (diluted 5X with sterile water) (W+OS) were applied. Leaves at the same position from untreated plants served as controls. Plants were grown in a glasshouse under 16/8 h (long day) (26-28°C) supplemental light from Master Suns-U PIA Agro 400-W sodium lights.

### Identification of miRNAs and precursors, and prediction of miRNA targets

To identify conserved miRNA and tasiRNA, we blasted all conserved plant miRNAs present in miRBase ( http://www.mirbase.org) against *N. attenuata*’s in house 454-transriptome database using default search parameters allowing 1 or 2 mismatches (Figure 1A). Hits of 20 to 24 nt sequences against non-coding transcripts with up to four nucleotide mismatches were selected as candidates to check for predicted miRNA secondary structure. The public web-based mFOLD server ( http://mfold.rna.albany.edu/) was used to predict secondary stem-and-loop structures using default parameters, folding temperature (37°C) and ionic conditions (1M NaCl) with minimum free energy (MFE) formations (Table 1). Predicted miRNA-precursors were additionally depicted using RNAshapes ( http://bibiserv.techfak.uni-bielefeld.de/rapidshapes) [60] to create stem-and-loop structures ( Additional file 1). For *TAS3* identification we used the following *TAS3* orthologs: *Saccharum officinarum* (EU327139), *Sorghum bicolor* (EU327137), *Zea mays* (EU327127, EU293143), *Oryza barthii* (GQ420228), *Triticum aestivum* (EU327134), *A. thaliana TAS3a* (AT3G17185), *TAS3b* (AT5G49615), *TAS3c* (At5g57735), *Nicotiana tabacum* (FJ804751, FJ804743), *Nicotiana benthamiana* (FJ804742), *Datura stramonium* (FJ804744). A neighbor-joining tree was built for *TAS3* transcripts using MEGA4 with group evaluation and 1000 bootstrap replicates [61].

### RNA extraction and RNA blot hybridization

RNA blot hybridization was performed as described by Molnar et al. (2007) [62]. Extracted total RNA was treated with DNase I (Fermentas; http://www.fermentas.com) according to the manufacturer’s protocol. Enzymes were removed by phenol-chloroform extraction and total RNA was re-isolated by ethanol precipitation. A denaturing 15% polyacrylamid gel containing 7M urea was prepared using the BIORAD Mini-Protean 3 Cell system ( http://www.bio-rad.com). Forty μg of total RNA were denatured with an equal volume of 2x gel-loading dye (Fermentas, http:/ http://www.fermentas.de) at 65°C for 5 minutes. Denatured RNA samples were loaded into the prerun 15% denaturing polyacrylamid gel at 50 V. A ZR small-RNA^TM^ Ladder (Zymo Research, http://www.zymoresearch.com) was also loaded. The gel was run at 150 V until the bromphenol blue in the loading dye reached the bottom of the gel. Next, the portion of the gel containing small RNA (between the bromphenol blue and xylene cyanol) was cut out and blotted onto a nylon membrane (Nytran Supercharge; http://www.whatman.com) by capillary wet transfer overnight. Transferred RNA was crosslinked to the membrane with UV light at 1200 μJ (UV Stratalinker 2400, Stratagene; http://www.stratagene.com). The membrane was pre-hybridized in 5–10 mL of ULTRAhyb-Oligo Hybridization Buffer ( http://www.ambion.com) at 40°C for at least 30 min. DNA oligo probes ( Additional file 2) were labeled using ^32^P with U4 polynucleotide kinase (Fermentas; http://www.fermentas.com). The reaction mix was incubated at 37°C for 1 h. Unincorporated nucleotides were separated using a Microspin G-25 column according to the manufacturer’s instructions (GE Healthcare, http://www.gelifesciences.com). The labeled probe was mixed with the hybridization solution and hybridized to the membrane at 40°C overnight. The membrane was washed with a washing solution (2xSSC, 0.1% SDS) at 40°C for 10 minutes, wrapped with plastic saran wrap, and exposed to phospho*-*imaging plates.

### Quantitative real-time PCR (qPCR) and cDNA microarray

One μg of total RNA were reverse-transcribed using SuperScript® II reverse transcriptase following the manufacturer’s protocol (Invitrogen; http://www.invitrogen.com). Twenty μg of cDNA was used to perform quantitative real-time PCR with SYBR Green using gene-specific primers ( Additional file 4 and 10) designed for *MIR* and target genes. Elongation factor (*NaEF*) was used as reference house-keeping gene for analysis.

For reverse transcription of miRNA and tasiRNAs into cDNA, we used the miScript Reverse Transcription Kit (Qiagen; http://www.qiagen.com). 10 μg of total RNA were used for qPCR ( Additional file 5) with the miScript SYBR Green PCR Kit (Qiagen; http://www.qiagen.com) to quantify miRNAs and tasiRNAs. All qPCR data were analyzed using the 2ΔΔCt calculation method [63].

We used a cDNA microarray NCBI GEO database (Platform GPL13527, accession number GSE30287) [46]. For data analyses, raw data was normalized to the 75th percentile and log2-transformed. Comparisons with greater than a 2-fold change were tested by Fisher’s PLSD test following an ANOVA.

### Phytohormone analysis

JA and JA-Ile were co-extracted from leaf tissue as previously described [64]. One hundred to 150 mg of lamina tissue from control and treated plants were used for phytohormone extraction with 1 ml of ethyl acetate spiked with 200 ng of D_2_-JA, and 40 ng of ^13^C_6_-JA-Ile as internal standards. Fifteen μL of the supernatant were analyzed on a Varian 1200 L Triple-Quadrapol-MS with a ProntoSIL column (C18; 5 μm, 50 × 2 mm).

### Statistical analysis

Data were calculated with the StatView Software using the one-way analysis of variance ANOVA (means were compared by the lowest standard deviation (LSD)) algorithm (SAS Institute Inc., Cary, NC, USA).

## Competing interests

The authors declare that they have no competing interests**.**

## Authors’ contributions

TAB performed the experimental work and analyzed the data. TAB, ITB and SGK participated in the design of the study. ITB and SGK conceived of the study and edited the manuscript. TAB drafted the manuscript. All authors read and approved the final manuscript.

## Supplementary Material

Additional file 1 **Stem-and-loop structures of identified miRNAs in*N. attenuata.*** Click here for file

Additional file 2 List of smRNA probes used for RNA blot hybridization. Click here for file

Additional file 3 **Accumulation of several miRNAs in rosette leaves of*N*.*attenuata*.** RNA blot hybridization performed to examine the accumulation of miRNAs in rosette leaves of *N*. *attenuata*. Ethidium bromide staining of rRNA is shown as a loading control. Click here for file

Additional file 4 List of primers used for qPCR analysis of primary miRNAs. Click here for file

Additional file 5 List of smRNA-specific forward primers used for miScript qPCR. Click here for file

Additional file 6 List of putative miRNA targets containing miRNA binding site. Click here for file

Additional file 7 Primer sequences of miRNA targets. Click here for file

Additional file 8 **Transcript abundance of miRNA target genes.** Abundance of miRNA targets after W+W and W+OS treatments. For each sample, one leaf on the rosette of a 32-day-old WT plant was left untreated (control) or treated with wounding plus water (W+W) or wounding plus OS (W+OS) and harvested 1 h or 5 h post treatment. Shown are mean (± SE) levels of three replicates per line. Letters indicate significant differences (*P* < 0.05) in Fisher’s PLSD tests following an ANOVA. Click here for file

Additional file 9 **JA and JA-Ile levels were impaired in ir-aoc plants after elicitation.** Accumulation of Jasmonic acid (JA) and jasmonoyl isoleucine (JA-Ile) increased in W+W and W+OS treated leaves and these accumulations were dramatically altered in ir-aoc plants. For each sample, one leaf on the rosette of a 32-day-old plant was treated and harvested 1 h or 5 h post treatment. Untreated plants served as control. Asterisks indicate significant differences (***, P < 0.001) in Fisher’ s PLSD tests following an ANOVA. Click here for file

Additional file 10 **qPCR data showing accumulation of primary miRNA transcripts in W+W and W+OS treated leaves in wild type and ir-*aoc.*** For each sample, one leaf on the rosette of a 32-day-old plants was treated with wounding plus water (W+W) or wounding plus OS (W+OS) and harvested 1 h or 5 h post treatment. Untreated plants served as control (CTRL). Shown are mean (± SE) levels of three replicates. Letters indicate significant differences (*P* < 0.05) in Fisher’s PLSD test following an ANOVA. Click here for file

Additional file 11 **Time course expression of miRNAs and their targets in W+W and W+OS treated leaves. (A)** qPCR data showing the abundance of mature miRNAs. **(B)** Microarray data showing the accumulation of miRNAs targets. For each sample, one leaf on the rosette of a 32-day-old WT plant was left untreated (control) or treated with wounding plus water (W+W) or wounding plus OS (W+OS) and harvested 1 h or 5 h post treatment. Shown are mean (± SE) levels of three replicates per line. Lowercase letters (W+OS) and italic letters (W+W) indicate significant differences (*P* < 0.05) in Fisher’s PLSD tests following an ANOVA. Click here for file

Additional file 12 **Microarray data showing accumulation of primary miRNAs in W+W and W+OS treated leaves of*N. attenuata*.** Mean (± SE) levels of normalized transcript abundance of miRNAs and tasiRNAs target genes after W+W and W+OS treatments. Untreated plants served as control (CTRL). Signal intensities in microarray data was normalized using the 75^th^ percentile value and log2-transformation. Letters indicate significant differences (*P* < 0.05) in Fisher’s PLSD test following an ANOVA. Click here for file
