# Supplementary figures and images for: Identification and profiling of miRNAs during herbivory reveals jasmonate-dependent and -independent patterns of accumulation in Nicotiana attenuata
Source: BMC Plant Biol. 2012 Nov 7;12:209. doi: 10.1186/1471-2229-12-209 (PMC3502350; doi:10.1186/1471-2229-12-209)

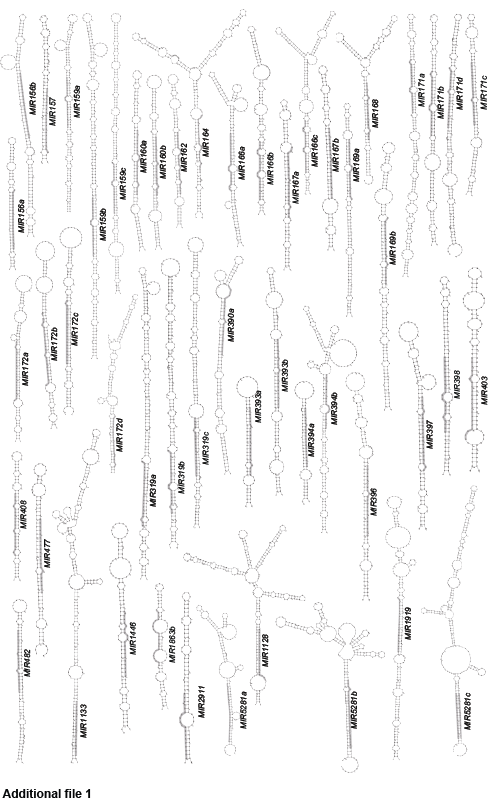

Supplement: Additional file 1 — Stem-and-loop structures of identified miRNAs inN. attenuata. [file 1471-2229-12-209-S1.tiff]

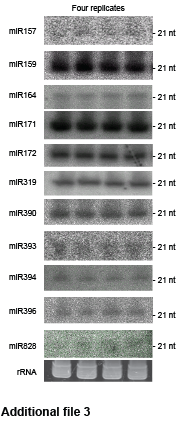

Supplement: Additional file 3 — Accumulation of several miRNAs in rosette leaves ofN.attenuata. RNA blot hybridization performed to examine the accumulation of miRNAs in rosette leaves of N. attenuata. Ethidium bromide staining of rRNA is shown as a loading control. [file 1471-2229-12-209-S3.tiff]

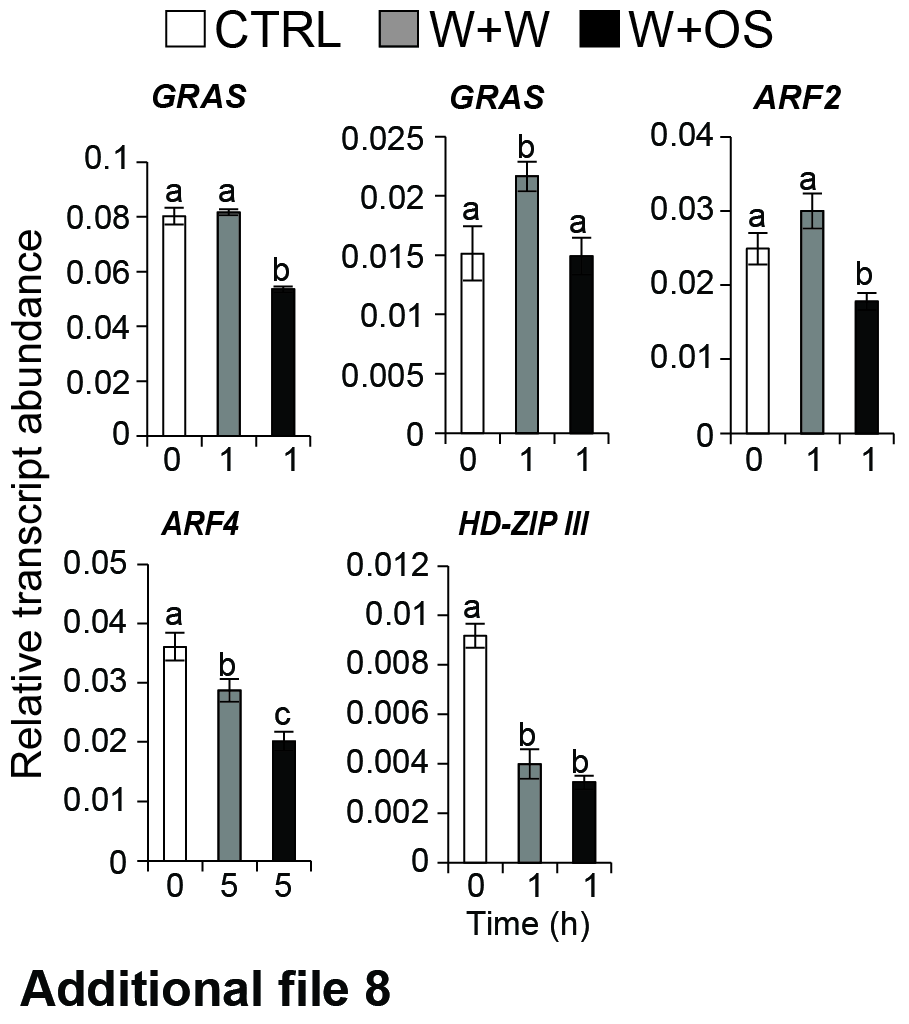

Supplement: Additional file 8 — Transcript abundance of miRNA target genes. Abundance of miRNA targets after W+W and W+OS treatments. For each sample, one leaf on the rosette of a 32-day-old WT plant was left untreated (control) or treated with wounding plus water (W+W) or wounding plus OS (W+OS) and harvested 1 h or 5 h post treatment. Shown are mean (± SE) levels of three replicates per line. Letters indicate significant differences (P < 0.05) in Fisher’s PLSD tests following an ANOVA. [file 1471-2229-12-209-S8.tiff]

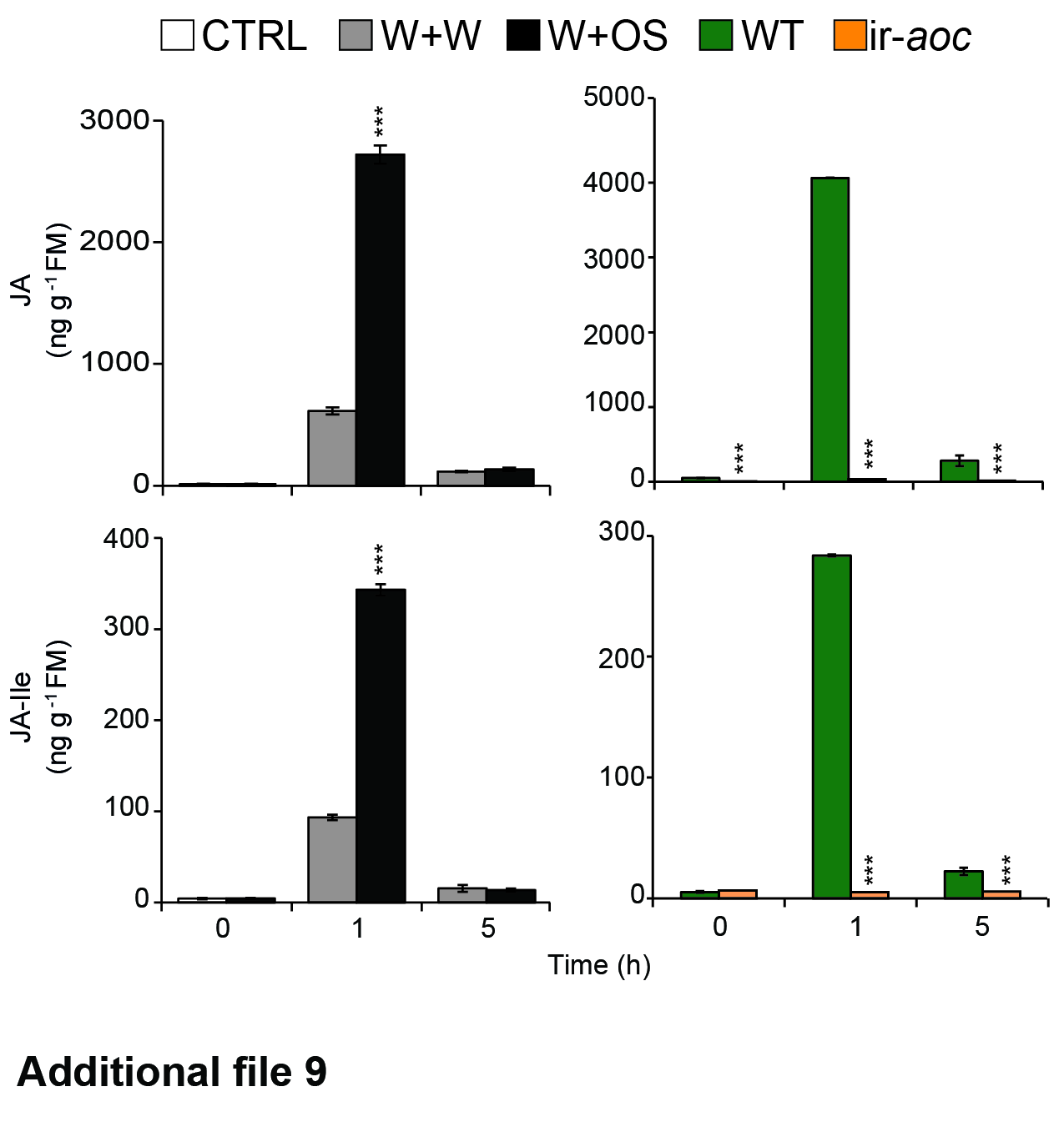

Supplement: Additional file 9 — JA and JA-Ile levels were impaired in ir-aoc plants after elicitation. Accumulation of Jasmonic acid (JA) and jasmonoyl isoleucine (JA-Ile) increased in W+W and W+OS treated leaves and these accumulations were dramatically altered in ir-aoc plants. For each sample, one leaf on the rosette of a 32-day-old plant was treated and harvested 1 h or 5 h post treatment. Untreated plants served as control. Asterisks indicate significant differences (***, P < 0.001) in Fisher’ s PLSD tests following an ANOVA. [file 1471-2229-12-209-S9.tiff]

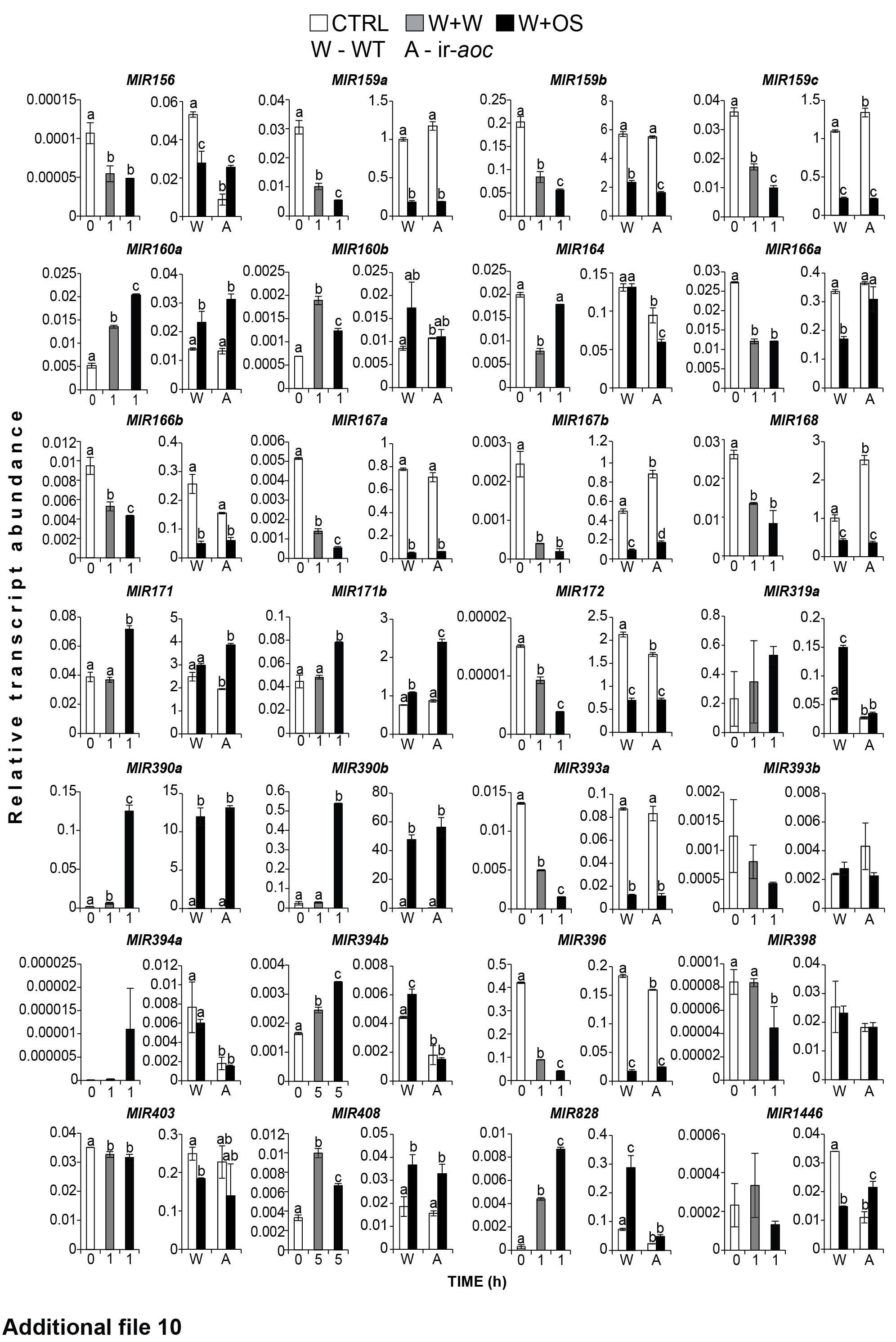

Supplement: Additional file 10 — qPCR data showing accumulation of primary miRNA transcripts in W+W and W+OS treated leaves in wild type and ir-aoc. For each sample, one leaf on the rosette of a 32-day-old plants was treated with wounding plus water (W+W) or wounding plus OS (W+OS) and harvested 1 h or 5 h post treatment. Untreated plants served as control (CTRL). Shown are mean (± SE) levels of three replicates. Letters indicate significant differences (P < 0.05) in Fisher’s PLSD test following an ANOVA. [file 1471-2229-12-209-S10.tiff]

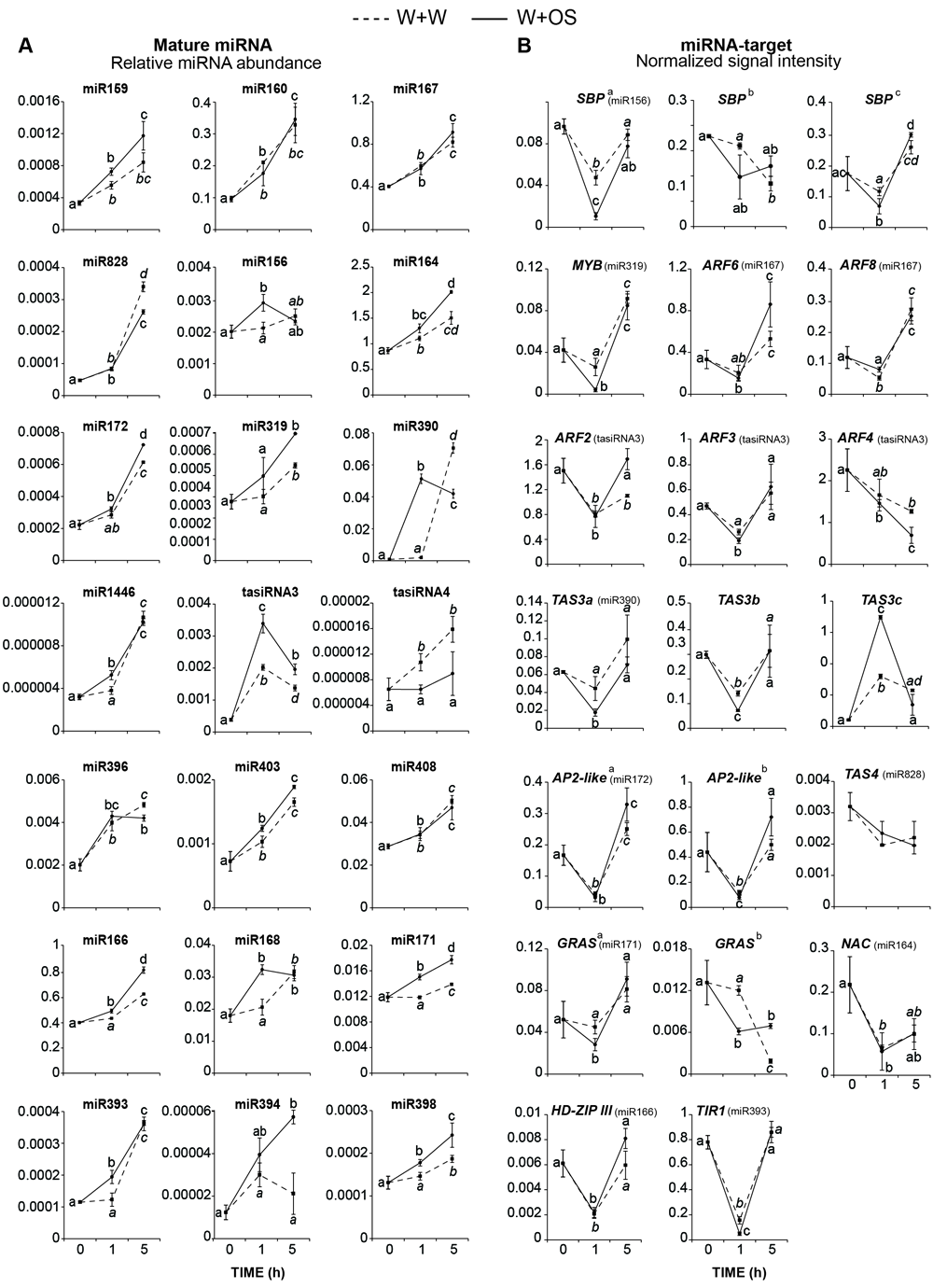

Supplement: Additional file 11 — Time course expression of miRNAs and their targets in W+W and W+OS treated leaves. (A) qPCR data showing the abundance of mature miRNAs. (B) Microarray data showing the accumulation of miRNAs targets. For each sample, one leaf on the rosette of a 32-day-old WT plant was left untreated (control) or treated with wounding plus water (W+W) or wounding plus OS (W+OS) and harvested 1 h or 5 h post treatment. Shown are mean (± SE) levels of three replicates per line. Lowercase letters (W+OS) and italic letters (W+W) indicate significant differences (P < 0.05) in Fisher’s PLSD tests following an ANOVA. [file 1471-2229-12-209-S11.tiff]

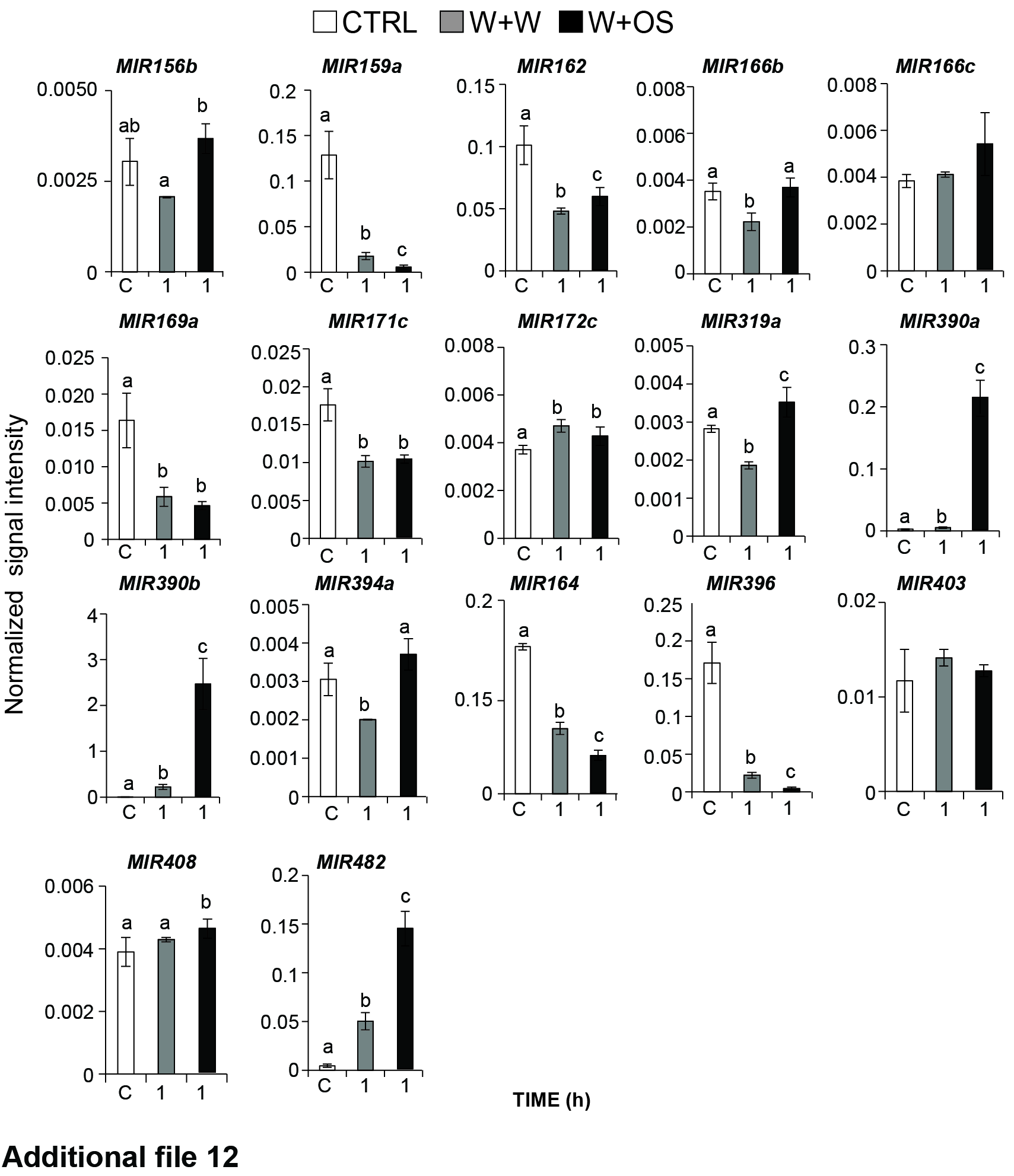

Supplement: Additional file 12 — Microarray data showing accumulation of primary miRNAs in W+W and W+OS treated leaves ofN. attenuata. Mean (± SE) levels of normalized transcript abundance of miRNAs and tasiRNAs target genes after W+W and W+OS treatments. Untreated plants served as control (CTRL). Signal intensities in microarray data was normalized using the 75th percentile value and log2-transformation. Letters indicate significant differences (P < 0.05) in Fisher’s PLSD test following an ANOVA. [file 1471-2229-12-209-S12.tiff]
