# Supplementary material for: Identification and profiling of miRNAs during herbivory reveals jasmonate-dependent and -independent patterns of accumulation in Nicotiana attenuata
Source: BMC Plant Biol. 2012 Nov 7;12:209. doi: 10.1186/1471-2229-12-209 (PMC3502350; doi:10.1186/1471-2229-12-209)
Supplement: Additional file 2 — List of smRNA probes used for RNA blot hybridization. [file 1471-2229-12-209-S2.rtf]

Additional file 2. List of smRNA probes used for RNA blot hybridization.

Primer	
Sequence
	

RM157-22	
GTTGCTCTCTATCTTCTGTCAA	
RM159-21	TAGAGCTCCCTTCAATCCAAA	
RM164-21	TGCACGTGCCCTGCTTCTCCA		
RM171-21	GATATTGGCACGGCTCAATCA	
RM172-23	ATGCAGCATCATCAAGATTCTCA	
RM319-21	AGGAGCTCCCTTCAGTCCAA	
RM390-21	GGTGCTATCCCTCCTGAGCTT	
RM393-22	GGATCAATGCGATCCCTTTGGA	
RM394-22	ATGGAGGTGGACAGAATGCCAA	
RM396-21	CTTCCCACAGCTTTATTGAAC	
RM828-22	TGGAATACTCATTTGAGCAAGA
	
