# Supplementary material for: Identification and profiling of miRNAs during herbivory reveals jasmonate-dependent and -independent patterns of accumulation in Nicotiana attenuata
Source: BMC Plant Biol. 2012 Nov 7;12:209. doi: 10.1186/1471-2229-12-209 (PMC3502350; doi:10.1186/1471-2229-12-209)
Supplement: Additional file 4 — List of primers used for qPCR analysis of primary miRNAs. [file 1471-2229-12-209-S4.rtf]

Additional file 4. List of primers used for qPCR analysis of primary miRNAs.

Primer	
Sequence	
Primer	
Sequence
	

PCR156A-PF-24	
ACAGAAGAGAGTGAGCACACATGG	
PCR319C-PF-24	
AGTAGCGGCAAGAGCTGTCATGT	
PCR156A-PR-24	AGTGTGACAGATAGAGAGCGAGCA	PCR319C-PR-24	AGTAGTTGCTCTCCCGTCATGCAA	
PCR159A-PF-26	GAGTATCTAACTGGTTTGTGGCTTGC	PCR390A-PF-24	GCGGTTATGTGGAAATTGCATGGG	
PCR159A-PR-25	GAAGAGAGATGAATGTAGAGCTCCC	PCR390A-PR-24	AAACTCAGGATGGATAGCGCAACG	
PCR159B-PF-26	ACCTATGGATCCCTCAGCCCTATCTA	PCR390B-PF-24	AAAGCTCAGGAGGGATAGCACCAT	
PCR159B-PR-25	GAAGAGAGATGAATGTAGAGCTCCC	PCR390B-PR-24	AGGCCATGAAACTCAGGATGGAGA	
PCR159C-PF-26	GAGTATCTAACTGGTTTGTGGCTTGC	PCR393A-PF-24	GTGGAGTATTCCAAAGGGATCGCA	
PCR159C-PR-25	GAAGAGAGATGAATGTAGAGCTCCC	PCR393A-PR-28	GGAGAAATCCGAAGAGATCGCATGATCC	
PCR160A-PF-24	ATACGTATATGCCTGGCTCCCTGT	PCR393B-PF-23	TGGTCTTCCCAGCAACTGAAGGA	
PCR160A-PR-24	TATGCTTGGCTCCTCATACGCCAT	PCR393B-PR-24	AGCTCCAAAGGATGAGTCCAAAGG	
PCR160B-PF-24	TGAGAAACTTGGATGTGCCTGGCT	PCR394A-PF-24	TTGGCATTCTGTCCACCTCCATTG	
PCR160B-PR-24	AATGTGGTATGCTTGGCTCCTTGC	PCR394A-PR-26	TTTGCAGAGCTAGTTTGGCACTCTGG	
PCR164-PF-24	AGCAGGGCACGTGCATTACTAACT	PCR394B-PF-24	TCTTTGGCATTCTGTCCACCTCCT	
PCR164-PR-29	GGGAAGACAGGCACATGAAGAACTAACTC	PCR394B-PR-24	TGGCAGTATGCCCACCTCCATAAT	
PCR166A-PF-24	GAGGAGAATGTTGTCTGGTTCGAG	PCR396-PF-25	CCACAGCTTTCTTGAACTGCATCTG	
PCR166A-PR-24	GGAATGAAGCCTGGTCCGAAATCA	PCR396-PR-24	CCCACAGCTTTATTGAACCGCAAC	
PCR166B-PF-24	GGGAATGTTGTTTGGCTCGAGGAT	PCR398-PF-24	CAGAGGAGTGAACATGGGAACACA	
PCR166B-PR-26	AATGAAGCCTGGTCCGACGATACCAA	PCR398-PR-24	GGTGACCTGAGAACACAAGTGCAT	
PCR166C-PF-24	GGAATGTTGTCTGGCTCGAGGAAT	PCR403-PF-24	CGTTTGTGCGTGATTCTGACAACC	
PCR166C-PR-21	AATGAAGCCTGGTCCGACGAC	PCR403-PR-24	GTGCGTGAATCTAACAAACACAACC	
PCR167A-PF-24	AGGTGATGCTGCCACATGATCTGA	PCR408-PF-25	AGAGGATAGACAGGGACGAGGTAGA	
PCR167A-PF-24	ACCACTAGTAGTTGAAGCTGCC	PCR408-PR-27	AGCCAGGGAAGAGGCAGTGCATAGAAT	
PCR167B-PF-24	ACACCACTATCAGTTGAAGCTGCC	PCR828-PF-24	CCTTTCTTGTGAAGGCCTCTTGCT	
PCR167B-PR-24	ACCAACTAACGGGTGAAGCTACCA	PCR828-PR-26	CTTCATGCCCAAATGAGTATCTCAAG	
PCR168-PF-24	ACTGAATCGGAGACTGCGGTGAAT	PCR1446-PF-24	TCTCTCCCTCAATGGCTGCTCATA	
PCR168-PR-24	ACTGTTACCACACAGCACAGCCTA	PCR1446-PR-24	CTGAACTCAATCTCTCATGGCTGT	
PCR171A-PF-24	GGTGCGGTTCAATGAGAAAGCAGT	PCRTAS3A-F-24	GTGCTCGAAGTCATGGTTCGTCTA	
PCR171A-PR-24	TTGCTACACGTGATATTGGCACGG	PCRTAS3A-R-24	AGCTCAGGAAAGGATAACACCGCA	
PCR171B-PF-24	GGTGCGGTTCAATGAGAAAGCAGT	PCRTAS3B-F-24	ACCTTGCAAGTCCGAGGTCTTCTT	
PCR171B-PR-24	TAGTGACACGTGATATTGGCACGG	PCRTAS3B-R-25	GCTCAGAAGGGATAGAAATGAGACG	
PCR172A-PF-26	GCAGCTGCAGCATCATCAAGATTCAC	PCRTAS3C-F-24	ACCTAGCCGAGTCTTCTTGACCTT	
PCR172A-PR-23	CAGCATCATCAAGATTCTCACG	PCRTAS3C-R-24	AGGAGAACATGAGTTGAGCGGGAA	
PCR319A-PF-24	AGGGATTTGGATTAGCTGCCGACT	PCRTAS4-PF-25	GGAGTATTGAGAAACAGAAGGGCCA	
PCR319A-PR-24	ACCTCCCGCATCATTCACACATTC	PCRTAS4-PR-26	GCTACATCATCCTCGTATCTTCATCG	
PCR319B-PF-24	ACACATGGGCGGTGATAAGGTTCA			
PCR319B-PR-24	ACTCCCGCTTCATTCAGTCAGCTA
			
