# Supplementary material for: Identification and profiling of miRNAs during herbivory reveals jasmonate-dependent and -independent patterns of accumulation in Nicotiana attenuata
Source: BMC Plant Biol. 2012 Nov 7;12:209. doi: 10.1186/1471-2229-12-209 (PMC3502350; doi:10.1186/1471-2229-12-209)
Supplement: Additional file 5 — List of smRNA-specific forward primers used for miScript qPCR. [file 1471-2229-12-209-S5.rtf]

Additional file 5. List of smRNA-specific forward primers used for miScript qPCR.

Primer	
Sequence
	

FM156-21	
TGACAGAAGAGAGTGAGCACA	
FM159-21	TTTGGATTGAAGGGAGCTCTA	
FM160-21	TGCCTGGCTCCCTGTATGCCA	
FM164-21	TGGAGAAGCAGGGCACGTGCA	
FM166-21	TCGGACCAGGCTTCATTCCCC	
FM167-21	TGAAGCTGCCAGCATGATCTA	
FM168-21	CCCGCCTTGCATCAACTGAAT	
FM171-21	TGATTGAGCCGTGCCAATATC	
FM172-23	TGAGAATCTTGATGATGCTGCAT	
FM319-21	TTGGACTGAAGGGAGCTCCCT	
FM390-21	AAGCTCAGGAGGGATAGCACC	
FM393-22	TCCAAAGGGATCGCATTGATCC	
FM394-22	TTGGCATTCTGTCCACCTCCAT	
FM396-21	GTTCAATAAAGCTGTGGGAAG	
FM398-21	TGTGTTCTCAGGTCACCCCTT	
FM403-21	TTAGATTCACGCACAAACTCG	
FM408-21	ATGCACTGCCTCTTCCCTGGC	
FM828-22	TCTTGCTCAAATGAGTATTCCA	
FM1446-20	TTCTGAACTCTCTCCCTCAA	
FMTAS3	TTCTTGACCTTGTAAGACCTTTT
MTTGACCTTGTAAGACCCC	
FMTAS4-22	AACCTCAACCTCGGACCTTCAT	
		
