# Supplementary material for: Identification and profiling of miRNAs during herbivory reveals jasmonate-dependent and -independent patterns of accumulation in Nicotiana attenuata
Source: BMC Plant Biol. 2012 Nov 7;12:209. doi: 10.1186/1471-2229-12-209 (PMC3502350; doi:10.1186/1471-2229-12-209)
Supplement: Additional file 7 — Primer sequences of miRNA targets. [file 1471-2229-12-209-S7.rtf]

Additional file 7. Primer sequences of miRNA targets.

Primer	
Sequence
	

PCRARF2-F-24	
TAACTGCAGACCCTTCACAAGCCA	
PCRARF2-R-22	CCACTGATGGTGGCCATAACAT	
PCRARF4-F-26	TGGACTTTGAGGAGTCGGTAAGATCC	
PCRARF4-R-24	TCCATAGGGAGGTGATATCAGACCTAA	
PCRHDZIP2-F-24	ACCATTTGACATCTCAGCATCCGC	
PCRHDZIP2-R-24	ACTCAACAGCAGTTCCAGTAGCCT	
PCRGRAS-F-24	GGACAAATGCGGCAGCAAGTCAAT	
PCRGRAS-R-24	CTGTTGTTGATGACGAGGCACCAT	
PCRGRAS2-F-24	CCTCGTCATCAACAGCAGGAACAA	
PCRGRAS2-R-26	GCGTTTGAGAATTGTCCGGCCAGTAA

	
